# Draft Genome Sequence of Pantoea sp. Strain MBLJ3, Isolated in a Laboratory Environmental Control Study

**DOI:** 10.1128/genomeA.01595-14

**Published:** 2015-02-26

**Authors:** Jing Zhang, Guo-Chiuan Hung, Haiyan Lei, Tianwei Li, Bingjie Li, Shien Tsai, Shyh-Ching Lo

**Affiliations:** Tissue Microbiology Laboratory, Division of Cellular and Gene Therapies, Office of Cellular, Tissue and Gene Therapies, Center for Biologics Evaluation and Research, Food and Drug Administration, Silver Spring, Maryland, USA

## Abstract

This report describes the draft genome sequence of a newly isolated strain, Pantoea sp. MBLJ3. The genome is 4.8 Mb in size, with a G+C content of 54.27%, and it contains 4,522 protein-coding sequences, 69 tRNA genes, and 5 rRNA genes.

## GENOME ANNOUNCEMENT

The genus Pantoea, a member of the *Enterobacteriaceae* family, comprises a large group of Gram-negative bacteria isolated from plants, soil, and water, as well as human diseased tissues (1–5). Many Pantoea sp. bacteria are phytopathogens. On the other hand, many nonpathogenic Pantoea spp. can promote growth in plants by promoting nitrogen fixation and acting as biofertilizers (6) or by producing plant growth hormones (7). Commercially, several Pantoea spp. have been developed into biological control agents to prevent fire blight disease in plants through the secretion of a variety of antibiotics (8–11).

In a routine environmental control study in our laboratory, we isolated a bacterium (Pantoea sp. strain MBLJ3) on a brain heart infusion (BHI) agar plate. The biochemical and metabolic characterization of the bacterium using Biolog’s microbial identification systems (Biolog, Inc., CA) indicated that the newly isolated microbe belongs to the genus Pantoea. The study of the 16S rRNA gene sequence of the bacterium revealed ~99% similarity to that of 3 different established species of Pantoea: Pantoea sp. strain αB, Pantoea vagans, and Pantoea ananatis.

The genomic sequencing of Pantoea sp. MBLJ3 was conducted using the Illumina MiSeq platform with 2 × 250-bp paired-end sequencing mode. After quality filtering, 6,847,708 reads were assembled into 37 contigs using the CLC Genomics Workbench version 6.0, for a total genome length of 4,821,318 bp (*N*_50_, 467,044 bp; average length, 41,635 bp; G+C content, 54.27%) and a genome coverage of 236×. The comparative analysis of the genome contents using progressiveMauve (12) revealed that Pantoea sp. MBLJ3 has dissimilarities of 12%, 18%, and 49% to the genomes of Pantoea sp. αB, P. vagans, and P. ananatis, respectively. This finding suggests that the newly isolated Pantoea sp. MBLJ3 might be a new Pantoea species.

The functional annotation was carried out by Rapid Annotations using Subsystems Technology (RAST) version 2.0 (13). The draft genome contains 4,598 coding regions (2,337 genes transcribed from the positive strand and 2,261 genes from the negative strand), of which 4,522 were annotated as protein-coding sequences and 74 were predicted as RNA genes (69 tRNA and 5 rRNA genes). Notably, the annotated genome has a subsystem containing colicin V and a bacteriocin production cluster of 8 genes. These genes are potentially involved in the synthesis of antibiotics to prevent the growth of competing microbes.

A recent study using multilocus sequence analysis showed that the phylogenetic relationships among the members of the Pantoea genus are quite diverse (14). The genome sequence of Pantoea sp. MBLJ3 reported here will allow comparative genome analysis of other members of the genus Pantoea and for further phylogenetic studies.

### Nucleotide sequence accession numbers.

This whole-genome shotgun project has been deposited in GenBank under the accession no. JSUT00000000. The version described in this paper is the first version, JSUT01000000.
